# Intestinal dysbiosis in critically ill patients: a case–control study of *Enterobacteriaceae* enrichment and reduced microbial diversity

**DOI:** 10.3389/fmed.2025.1680262

**Published:** 2025-11-27

**Authors:** Dan Wang, Jinman Li, Lei Wang, Heng Tian, Xuan Zhou, Xiaolei Wang, Huiyu Tian

**Affiliations:** The First Hospital of Hebei Medical University, Shijiazhuang, Heibei, China

**Keywords:** gut microbiome, dysbiosis, ICU, *Enterobacteriaceae*, sepsis, 16S rRNA sequencing

## Abstract

**Introduction:**

Critical illness disrupts gut microbiota homeostasis, potentially exacerbating systemic inflammation and adverse outcomes. This study investigates gut dysbiosis patterns in ICU patients, with a focus on *Enterobacteriaceae* enrichment and microbial diversity loss, to identify biomarkers and therapeutic targets.

**Methods:**

In this case–control study, 37 ICU patients (sepsis: *n* = 17; non-sepsis: *n* = 20) and 20 healthy controls were enrolled. Fecal samples underwent 16S rRNA sequencing (V3–V4 regions). Microbial diversity (Shannon/Simpson indices), <mark>beta diversity (Bray–Curtis PCoA)</mark>, and taxonomic differences (LEfSe, LDA > 2.5) were analyzed using QIIME2 and R.

**Results:**

Critically ill patients showed reduced alpha diversity vs. controls (Shannon *p* = 0.04; Simpson *p* = 0.04). *Enterobacteriaceae* (phylum *Proteobacteria*) were significantly enriched in ICU patients (LDA = 4.2, *p* < 0.01), while *Ruminococcus* dominated controls. Beta diversity differed markedly (PERMANOVA *R*^2^ = 0.199, *p* = 0.001). No diversity differences were observed between sepsis/non-sepsis subgroups (*p* > 0.05).

**Conclusion:**

ICU patients exhibit gut dysbiosis characterized by *Enterobacteriaceae* expansion and diversity loss, independent of sepsis status. These findings underscore the gut microbiome’s role in critical illness and support exploring microbiota-targeted interventions (e.g., selective probiotics) to improve outcomes.

## 1 Introduction

Critically ill patients, including those with conditions such as sepsis, trauma, and respiratory failure requiring intensive life-support interventions in the intensive care unit (ICU), frequently experience multiple organ dysfunction, extreme physiological stress, and heightened vulnerability to nosocomial infections, all of which contribute to elevated mortality rates (1–3). Among the many organ systems impacted by critical illness, emerging evidence points to significant ecological and functional disruption of the intestinal microbiota (4). The gut microbiota consists of trillions of microbes coexisting symbiotically within the human gastrointestinal system, and it plays a fundamental role in maintaining host physiology (5). A diverse and stable microbial community supports gastrointestinal homeostasis, nutrient metabolism, immune modulation, and systemic health (6). Studies have identified 1,000 of distinct bacterial species within the gut ecosystem, many of which perform essential functions for host survival (7). Dysbiosis entails alterations in the composition, diversity, and abundance of the microbiota, and can impair these functions and contribute to disease pathogenesis (8). Gut dysbiosis has been implicated in a broad spectrum of pathological conditions, including infections, inflammatory bowel disease, metabolic disorders, and malignancies (9). In the context of critical illness, the intestinal microbiota undergoes profound shifts characterized by a decline in microbial diversity and a dominance of pathogenic taxa such as *Enterobacteriaceae* (10). Increasing evidence suggests that microbial components or metabolites may translocate into the systemic circulation, thereby influencing immune responses and contributing to disease severity (11).

Critically ill individuals often require comprehensive organ support and continuous monitoring due to their unstable physiological status and increased risk of adverse outcomes (12). Despite extensive investigations into critical illness, relatively few studies have focused on the gut microbiota in this patient population. In this study, we compared the intestinal microbial profiles of ICU patients with those of healthy individuals and observed a marked reduction in microbial richness and diversity among the critically ill. Sepsis, a life-threatening complication frequently encountered in ICU settings, remains a global clinical challenge despite extensive research aimed at reducing its mortality burden. Timely identification of patients at risk for sepsis and its associated complications is critical for improving outcomes (13). Although various biomarkers such as C-reactive protein (CRP), white blood cell count (WBC), procalcitonin (PCT) levels, platelet count, and serum lactate have been proposed for early sepsis prediction, their utility in clinical practice remains limited (14, 15). Consequently, novel biomarkers and predictive tools are urgently needed (16). While previous studies have established the occurrence of gut dysbiosis in critically ill patients, many have focused on specific subpopulations, such as septic patients, or have not directly compared the dysbiosis patterns between septic and non-septic critically ill individuals within the same cohort. This study aims to characterize the gut microbiome in a general ICU cohort and to determine whether the dysbiosis pattern is driven primarily by the presence of sepsis or is a common feature of critical illness itself. By doing so, we seek to identify core alterations in the gut ecosystem that are fundamental to critical illness, which could serve as more universal targets for future microbiota-directed interventions.

## 2 Materials and methods

### 2.1 Study design

This case–control study enrolled 37 critically ill subjects admitted to the First Department of Critical Care Medicine at Hebei Medical University First Hospital between January 2023 and September 2024. Additionally, 20 healthy volunteers were recruited as control participants. Informed consent was obtained from all participants or their legal representatives under clinician supervision. The study received approval from the local institutional ethics committee. Patients under 18 years of age were excluded, as were individuals who were pregnant or breastfeeding. The study was approved by the Ethics Committee of The First Hospital of Hebei Medical University (Approval No. V1.0/20250801). As an exploratory, hypothesis-generating study, a formal *a priori* sample size calculation was not conducted. The sample size was determined based on feasibility and patient recruitment over the defined study period (January 2023–September 2024).

### 2.2 Data collection

Demographic, clinical, and laboratory parameters, including age, sex, primary diagnosis, ICU length of stay, mechanical ventilation duration, CRP, and PCT levels, were recorded upon ICU admission. Severity of illness was assessed within 48 h using the Sequential Organ Failure Assessment (SOFA) and Acute Physiology and Chronic Health Evaluation II (APACHE II) scoring systems. Fecal samples were collected at a single time point within 48 h of ICU admission. In cases where stool was unavailable, rectal swabs were obtained as an alternative. This study design provides a cross-sectional snapshot of the gut microbiota at ICU admission and does not capture longitudinal dynamics. All samples were immediately stored at −80 °Cfor downstream analysis.

### 2.3 Gut microbiota analysis

Samples were collected in accordance with standardized procedures by trained medical personnel and delivered to the microbiology research laboratory within 24 h. Upon arrival, samples were homogenized, aliquoted, and preserved at −80 °C until DNA extraction. Microbial DNA was isolated using the MP FastDNA Spin Kit for Feces (MP Biomedicals, Santa Ana, CA, USA), following the manufacturer’s protocol. The V3–V4 hypervariable regions of the 16S rRNA gene were amplified using primers 314F (CCTAYGGGRBGCASCAG) and 806R (GGACTACNNGGTATCTAAT). Amplicons were confirmed by 1.5% agarose gel electrophoresis and purified using magnetic bead-based methods (Shanghai Yasen Biotechnology Co., Ltd.). Paired-end sequencing (PE250) was conducted on the Illumina NovaSeq6000 platform. Post-sequencing, reads were filtered for chimeras using VSEARCH v2.10.3 (17). Operational taxonomic units (OTUs) were clustered using QIIME (18) with a 97% similarity threshold via UCLUST (19). Taxonomic assignments were compared to SILVA 132 for validation.

### 2.4 Data analysis

Data were analyzed in SPSS 27.0. Continuous variables were reported as mean ± standard deviation (x̄ ± s) if they conformed to a normal distribution and compared using independent sample *t*-tests. Skewed variables were presented as medians with interquartile ranges [M (P25, P75)] and compared using the Wilcoxon rank-sum (Mann–Whitney *U*) test. Categorical data were expressed as counts and percentages and assessed using the chi-square (*χ*^2^) test. Correlations were assessed based on Spearman’s rank correlation coefficient. A *p* < 0.05 was deemed significant.

Alpha diversity metrics including Shannon-Wiener index, Simpson’s index, Chao1 richness estimator, and phylogenetic diversity (PD) were calculated using QIIME2 to evaluate microbial richness and evenness. Rank-abundance curves were plotted to assess sequencing depth and distribution. Differences in alpha diversity indices (Shannon and Gini–Simpson indices) between patient groups were assessed using the Wilcoxon rank-sum test (for two-group comparisons) via the ggpubr package in R. The *p*-values were derived from these non-parametric tests, which do not assume normal distribution of the data. Beta diversity was assessed via Principal Coordinates Analysis (PCoA) based on Bray–Curtis dissimilarity, using R software (v4.x.x) and the vegan package. The Bray–Curtis index was selected as it quantifies the compositional dissimilarity between samples based on the relative abundances of operational taxonomic units (OTUs). This makes it particularly effective for capturing community shifts driven by the expansion of dominant taxa and the suppression of others, which aligns with our hypothesis regarding dysbiosis in critical illness (characterized by the bloom of *Enterobacteriaceae* and the loss of anaerobic commensals). Differences in microbial community structure between groups were evaluated via PERMANOVA based on the Bray–Curtis distance matrix using the adonis2 function. Linear Discriminant Analysis Effect Size (LEfSe) was employed to identify taxa with significant intergroup differences. The LEfSe analysis was conducted in R using the microeco package (v0.10.0). Biomarkers were defined as taxa with LDA scores >2.5 and *p* < 0.05.

## 3 Results

### 3.1 General participant characteristics

A total of 37 critically ill patients were included in the study, comprising 17 individuals diagnosed with sepsis and 20 without sepsis. Additionally, 20 healthy volunteers served as controls, with a mean age of 37.2 years (range: 25–52 years). Age and sex distributions were comparable in the sepsis and non-sepsis patient groups. However, inflammatory biomarkers differed markedly between these two subgroups. Median procalcitonin (PCT) levels were significantly elevated in septic patients [15.27 (5.24, 33.84) ng/mL] compared to non-septic patients [1.65 (0.33, 4.47) ng/mL]. Similarly, C-reactive protein (CRP) levels were significantly higher in the sepsis group [152.18 (14.8, 171.22) mg/L] than in the non-sepsis group [51.43 (19.85, 75.27) mg/L] Table 1.

**Table 1:** Characteristics of critically ill patients.

Variables	Sepsis group (*n* = 17)	Non-sepsis group (*n* = 20)	*p*-value
Age	45.76 (66)	67.5 (53, 73)	0.98
Gender			0.17
Male	8 (0.47)	14 (0.7)	
Female	9 (0.53)	6 (0.3)	
APACHE II	16 (13.5, 20.0)	14.5 (11.5, 18.8)	0.29
SOFA	9 (7.5, 10.5)	8 (7.3, 10.0)	0.34
CRPFi	152.18 (14.8, 171.2)	51.43 (19.9, 75.3)	0.004
PCT	15.27 (5.2, 33.8)	1.65 (0.3, 4.5)	0.04
Length of stay in ICU	10 (5.0, 17.5)	10 (7.0, 29.8)	0.54
MV time	7 (3.5, 11.5)	4 (1.0, 11.8)	0.39

### 3.2 Microbiota profiling

**Figure 1:**
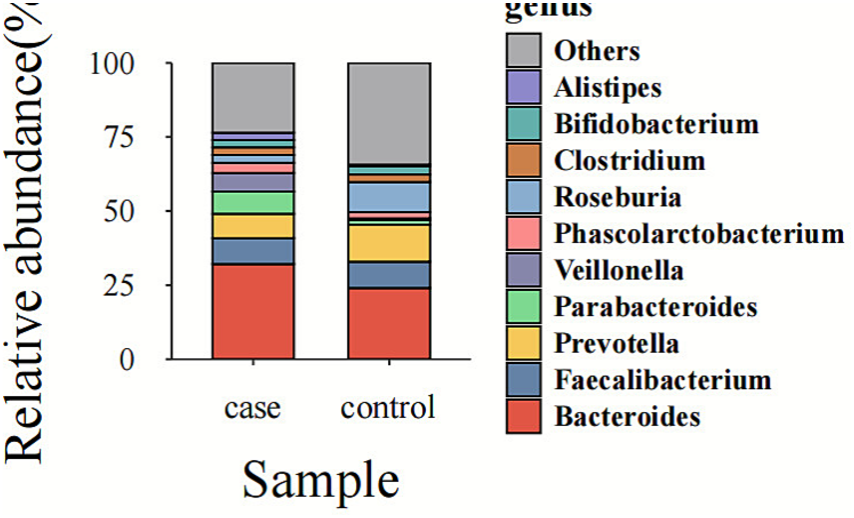
Bacteroides and Parabacteroides were more abundant in critically ill patients, while *Ruminococcus* showed greater abundance in healthy individuals.

The processing of raw 16S rRNA gene sequencing data was performed in the QIIME2 pipeline with the Casava1.8 paired-end format. After filtering and removal of chimeric sequences targeting the V3–V4 region, a total of 22,883,429 sequences were retained across 57 samples. These included 4,236,775 sequences from critically ill patients and 18,646,654 from healthy controls. Sequencing depth per sample ranged from 16,478 to 198,727 reads among critically ill patients and from 412,055 to 2,416,265 reads among healthy controls following DADA2 denoising. At the genus level, *Bacteroides* and *Parabacteroides* were more abundant in critically ill patients, whereas *Ruminococcus* was notably more prevalent in healthy individuals (Figure 1).

### 3.3 Microbial richness and diversity

**Figure 2:**
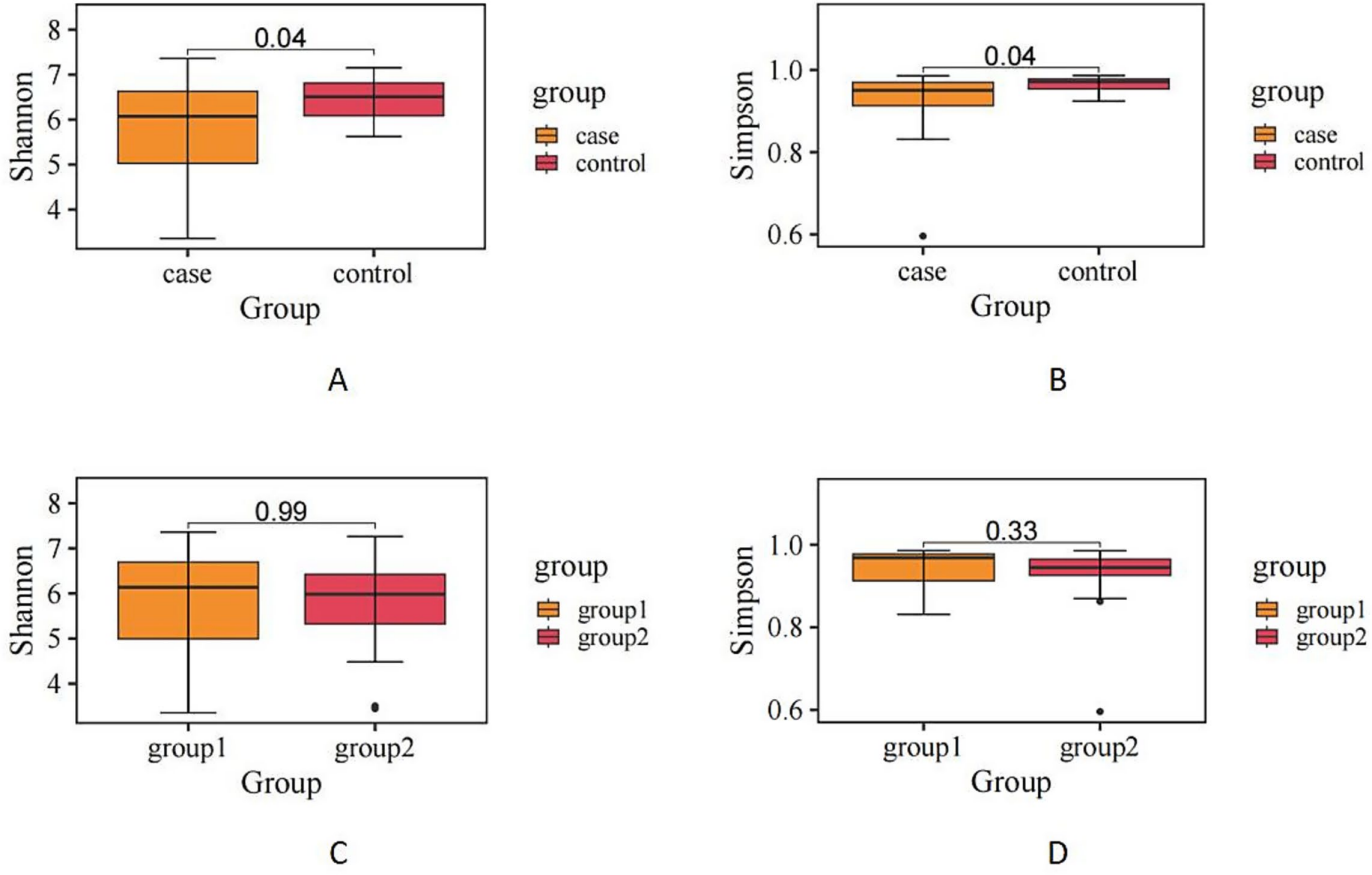
**(A,B)** There is a significant difference in alpha diversity between critically ill patients and healthy patients. **(C,D)** There is no difference in alpha diversity between patients with sepsis and those without sepsis.

Alpha diversity analysis revealed a statistically significant reduction in microbial diversity among critically ill patients compared to healthy controls, as indicated by both the Shannon index (*p* = 0.04) and the Gini–Simpson index (*p* = 0.04). However, when comparing the sepsis subgroup with non-septic critically ill patients, no significant differences in alpha diversity were noted (Shannon index *p* = 0.99; Gini–Simpson index *p* = 0.33) (Figure 2).

**Figure 3:**
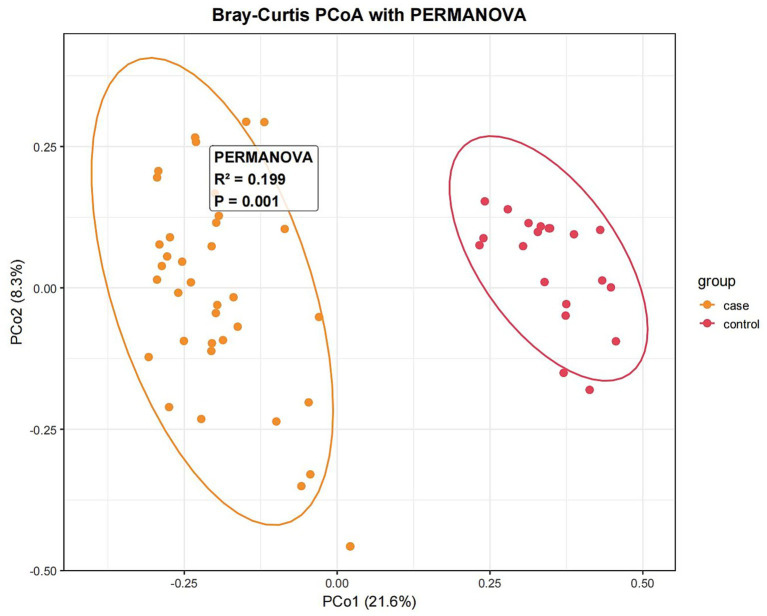
Principal coordinates analysis (PCoA) plot based on Bray–Curtis dissimilarity illustrating the separation of gut microbial communities between critically ill patients and healthy controls. The statistical significance of the group separation was confirmed by PERMANOVA (*R*^2^ = 0.199, *p* = 0.001).

Beta diversity was assessed using principal coordinates analysis (PCoA) based on Bray–Curtis distances. The microbial community structure differed significantly between critically ill patients and healthy controls (PERMANOVA *R*^2^ = 0.199, *p* = 0.001), consistent with reduced diversity in severe cases (Figure 3).

**Figure 4:**
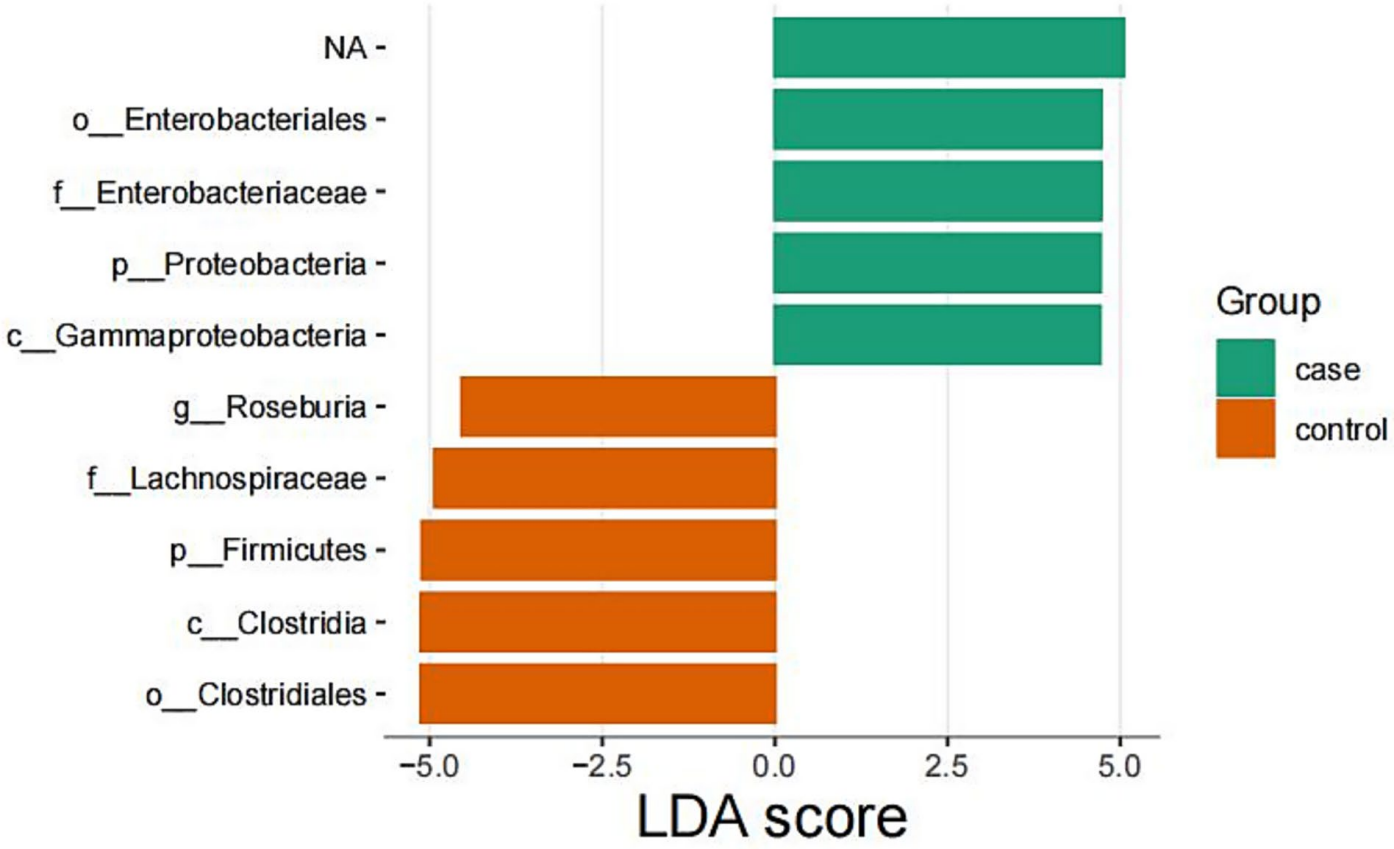
Members of the phylum *Proteobacteria*, particularly the family *Enterobacteriaceae*, were significantly enriched in critically ill patients. In contrast, thick-walled bacilli were significantly enriched in healthy patients.

LEfSe analyses identified microbial taxa that were differentially enriched between groups, with an LDA score threshold of >2.5 and *p* < 0.05. Notably, members of the phylum *Proteobacteria*, particularly the family *Enterobacteriaceae*, were significantly enriched in critically ill patients. In contrast, thick-walled bacilli were significantly enriched in healthy patients (Figure 4).

## 4 Discussion

In this case–control study profiling the gut microbiota of critically ill patients, we made two principal observations: first, critically ill patients exhibit a significant loss of microbial diversity and a distinct compositional shift characterized by the enrichment of *Enterobacteriaceae* compared to healthy controls; second, and perhaps more notably, this dysbiotic pattern was remarkably consistent between septic and non-septic ICU patients, suggesting it may be a hallmark of critical illness per se, rather than being specific to sepsis. Critical illness is often accompanied by substantial physiological derangements, which can profoundly affect the composition and stability of the intestinal microbiota. The gut microbiome plays a pivotal role in regulating metabolic and immune functions, and disturbances in this complex ecosystem during critical illness may significantly influence patient outcomes (20). Our study demonstrated that critically ill patients exhibit marked reductions in microbial diversity and shifts in community composition compared to healthy individuals. These findings are in line with the observations reported by Cho et al. (21), reinforcing the view that the gut microbiota undergoes substantial alterations in critically ill states. In particular, we observed a significant enrichment of *Enterobacteriaceae* in ICU patients. While typically present at low abundance in the healthy gut and generally non-pathogenic under homeostatic conditions, taxa such as Enterococcus, Staphylococcus, and members of the *Enterobacteriaceae* family have been linked to pathogenic activity during dysbiosis (22, 23). The overrepresentation of *Enterobacteriaceae* in the present patient cohort suggests that they may play a role in promoting inflammatory responses and infection severity, in line with prior evidence of their inflammatory potential (24).

Interestingly, while overall microbial diversity was reduced in critically ill patients, we found no statistically significant difference between septic and non-septic subgroups. This lack of distinction may be attributable to the widespread and often unregulated use of broad-spectrum antibiotics and other clinical interventions in the ICU, including sedatives, opioids, proton-pump inhibitors, and other agents known to alter the gut microbiome and their metabolites (25). The pronounced enrichment of *Enterobacteriaceae*, a family of facultative anaerobic bacteria, in our ICU cohort aligns with the established paradigm of gut dysbiosis in stress states (9). Critical illness, through factors like systemic inflammation, oxygen deprivation (tissue hypoxia), and nutrient availability, creates an intestinal environment that favors facultative anaerobes over obligate anaerobes, which constitute the majority of a healthy microbiota. This shift is mechanistically significant. *Enterobacteriaceae* are potent inducers of pro-inflammatory responses and can compromise intestinal barrier integrity. Their expansion can further perpetuate a cycle of inflammation and barrier dysfunction, potentially facilitating bacterial translocation and exacerbating systemic inflammatory response syndrome (SIRS) or even leading to secondary infections (20). Therefore, the observed *Enterobacteriaceae* bloom is not merely a biomarker of illness but may actively contribute to the pathophysiology and adverse outcomes in the ICU.

This study has several limitations that should be considered when interpreting the results. First, the sample size was relatively small and originated from a single center, which limits the statistical power, particularly for subgroup analyses such as the comparison between septic and non-septic patients. To provide a quantitative assessment of this limitation, a post-hoc sample size estimation was conducted. Based on the observed effect size (Cohen’s *d* ≈ 0.8) for the difference in the Shannon index between critically ill patients and healthy controls, a future study would require approximately 26 participants per group to achieve 80% power with a two-sided alpha of 0.05. Detecting more subtle effects within patient subgroups would necessitate an even larger cohort. Therefore, our findings, particularly those related to the lack of significant differences between subgroups, should be interpreted with caution and require validation in larger, multi-center studies. Second, the near-universal antibiotic exposure in our cohort represents a significant confounding factor that must be considered when interpreting our findings. While this reflects real-world clinical practice in the ICU setting, it precludes definitive conclusions about the independent effects of critical illness versus antibiotic exposure on gut microbiota alterations. The consistent dysbiosis pattern observed across both patient subgroups, despite the presence of two antibiotic-naive patients in the non-sepsis group, suggests a contribution of critical illness itself to microbial disruption. However, future studies with larger antibiotic-naive cohorts are needed to disentangle these complex interactions. Third, while we documented a profound dysbiosis, the 16S rRNA sequencing approach provides limited functional insights. Future studies utilizing shotgun metagenomics and metabolomics would be valuable to elucidate the functional consequences of the observed community shifts. Finally, although the use of antibiotics is nearly universal in the ICU and is a known major driver of dysbiosis, we did not stratify our analysis based on specific antibiotic regimens, which could have influenced the microbial patterns observed. Prospective, multi-center longitudinal studies that incorporate detailed medication data are needed to validate and extend our findings.

## 5 Conclusion

In summary, this study reveals that critically ill patients exhibit significant gut microbiota dysbiosis characterized by reduced microbial diversity and a notable enrichment of *Enterobacteriaceae*. These alterations may contribute to infection severity and adverse outcomes in ICU patients. Maintenance or restoration of gut microbial homeostasis could play an important role in mitigating disease progression. Therefore, targeting gut dysbiosis through microbiome-based diagnostics, probiotic interventions, or fecal microbiota transplantation may represent a promising avenue for future therapeutic strategies in critical care medicine.

## Data Availability

The datasets presented in this study can be found in online repositories. The names of the repository/repositories and accession number(s) can be found in the article/Supplementary material.
