# Supplementary material for: Intestinal dysbiosis in critically ill patients: a case–control study of Enterobacteriaceae enrichment and reduced microbial diversity
Source: Front Med (Lausanne). 2025 Nov 27;12:1680262. doi: 10.3389/fmed.2025.1680262 (PMC12695524; doi:10.3389/fmed.2025.1680262)
Supplement: Supplementary file 2 [file Table_2.DOCX]

| Medication Category | Sepsis Group (n=17) | Non-sepsis Group (n=20) | p-value |
| --- | --- | --- | --- |
| Any Antibiotic Use, n (%) | 17 (100%) | 18 (90%) | 0.492 |
| Antibiotic Duration, days | 10 (7-14) | 8 (5-11) | 0.152 |
| Common Antibiotic Classes, n (%) |  |  |  |
| Carbapenems | 10 (58.8%) | 8 (40.0%) | 0.267 |
| Glycopeptides | 9 (52.9%) | 7 (35.0%) | 0.278 |
| Cephalosporins | 6 (35.3%) | 9 (45.0%) | 0.551 |
| Other Medications, n (%) |  |  |  |
| Proton-Pump Inhibitors | 15 (88.2%) | 16 (80.0%) | 0.500 |
| Vasopressors | 14 (82.4%) | 12 (60.0%) | 0.138 |
| Sedatives | 13 (76.5%) | 14 (70.0%) | 0.661 |
